# iTRAQ-Based Proteomics Reveals the Potential Mechanisms Underlying Diet Supplementation with Stevia Isochlorogenic Acid That Alleviates Immunosuppression in Cyclophosphamide-Treated Broilers

**DOI:** 10.3390/ani16010025

**Published:** 2025-12-22

**Authors:** Jiatong Jin, Shuqi Zhao, Pengyu Zhao, Yushuo Zhang, Lifei Wu, Liangfu Zhou, Yasai Sun, Wen Zhao, Qian Zhou

**Affiliations:** 1 College of Food Science and Technology, Hebei Agricultural University, Baoding 071001, China; jinjiatong0127@163.com (J.J.);; 2 Chen Guang Biotechnology Group Co., Ltd., Handan 057250, China

**Keywords:** stevia isochlorogenic acid, immune organ indices, histological examination, proteomics, quantitative PCR, molecular docking analysis

## Abstract

The extensive use of antibiotics in intensive farming weakens immunity and threatens food safety. Stevia isochlorogenic acid (SICA), a kind of dicaffeoylquinic acid derived from stevia residue, exhibits strong antioxidant activity. This study evaluated the ability of SICA to improve immune function in an immunosuppressed broiler model. SICA significantly increased the spleen, thymus, and bursa of Fabricius indices (*p* < 0.05), alleviated spleen damage, and elevated serum interleukin-2 (IL-2), IL-4, interferon-γ, IL-1β, tumor necrosis factor-α, immunoglobulins (IgA, IgM, IgG), and complement components C3 and C4 (*p* < 0.05). Isobaric tags for relative and absolute quantification-based proteomics indicated that SICA enhanced splenic immune function by activating cell adhesion molecules, phagosomes, and the intestinal immune network for IgA production pathways. Quantitative PCR analysis showed upregulation of mRNA and protein levels of B-cell receptor, major histocompatibility complex class II, protein tyrosine phosphatase receptor type C, and neutrophil cytosolic factor 2 (p67phox) and downregulation of C-C motif chemokine receptor 9. Molecular docking demonstrated the strongest binding affinity between SICA and p67phox. Overall, SICA effectively alleviated immunosuppression in broiler chickens and represents a promising natural alternative to antibiotic feed additives.

## Simple Summary

Overuse of antibiotics in poultry farming can weaken broiler immune systems, increasing disease susceptibility and threatening food safety. Safe natural alternatives are therefore essential for supporting immune function. In this study, we investigated the immunomodulatory effects of stevia isochlorogenic acid (SICA) in an immunosuppressed broiler model. The results showed that SICA repaired damaged immune organs and increased the levels of key immune molecules in the blood, which are critical for effective disease resistance. Our analyses further indicated that SICA may exert its effects by activating the body’s innate defense pathways, enhancing immune cell communication, and improving the capacity to eliminate harmful pathogens. In summary, SICA effectively restored immunity in broilers through multiple coordinated mechanisms. These findings suggest that SICA could serve as a valuable natural feed additive to help farmers raise healthier broilers and reduce antibiotic dependence.

## 1. Introduction

As an important source of animal-derived food, chickens have many advantages. For instance, they can provide low-fat, high-quality protein and essential trace elements [1]. According to statistics from the Food and Agriculture Organization of the United Nations (FAO), in 2023, the global demand for chicken increased by 2.9 million metric tons per year between 2013 and 2023, promoting the rapid development of broiler farming [2]. Currently, intensive farming has replaced the conventional free-range mode, leading to a severe decline in the immunity of broilers [3], followed by a series of health issues, such as decreased immune function, slow growth and development, poor mental state, reduced resistance, and even death [3]. Consequently, antibiotics have long been used to prevent and improve the aforementioned situation. However, the overuse of antibiotics can lead to the emergence of antibiotic-resistant bacteria, resulting in significant food safety concerns [4]. Therefore, many countries have restricted the use of antibiotics to reduce the harm caused by their abuse and maintain the safety of animal-derived foods and public health [5]. Consequently, various bioactive compounds with significant immunomodulatory effects, including polyphenols, polysaccharides, microecological agents, enzymes, and acidifiers, have been examined [6,7].

Isochlorogenic acid compounds are phenolic acids composed of two caffeic acid moieties esterified with one quinic acid, with three major isomers: 3,5-di-O-caffeoylquinic acid (3,5-diCQA, isochlorogenic acid A), 3,4-di-O-caffeoylquinic acid (3,4-diCQA, isochlorogenic acid B), and 4,5-di-O-caffeoylquinic acid (4,5-diCQA, isochlorogenic acid C) [5]. These compounds are found in various plants, including vegetables, fruits, and traditional Chinese medicines [5]. Among these sources, stevia, a herbaceous plant of the Stevia genus in the Asteraceae family, is a highly nutritious medicine-food homolog and the main source for extracting steviol glycosides in the modern food industry [8]. The remaining stevia residue is often incinerated or landfilled, resulting in substantial resource waste [8]. In our previous studies, the content of stevia residue isochlorogenic acid (SICA) was ≥50%, and it consisted mainly of isochlorogenic acid A, isochlorogenic acid B, and isochlorogenic acid C.

The immune system is a network that comprises many components that function cooperatively, including various immune organs, immune cells, and immune molecules. However, when immune homeostasis is disrupted, the body can develop diseases such as allergic reactions, malnutrition, and autoimmune disorders [9]. The spleen is the largest lymphatic organ in the body and plays a crucial role in host defense. It initiates immune responses to blood-borne antigens, captures them, and distributes them to specific zones occupied by T and B cells [10]. It also filters foreign substances, removes aging or damaged red blood cells from circulation, and produces and releases hematological immune factors, such as immunoglobulins [11].

Previous studies have demonstrated the immunomodulatory effects of isochlorogenic acid compounds. Isochlorogenic acid A at a dose of 400 μg/mL can enhance the vitality of RAW 264.7 murine macrophage-like cells (RAW264.7 cells), inhibit the expression of NO, tumor necrosis factor-α (TNF-α), interleukin-6 (IL-6), toll-like receptor 2 (TLR2), and toll-like receptor 4 (TLR4) in inflammatory cells, and play a crucial role in regulating cellular immune responses and inflammation [5]. Zhou et al. [12] demonstrated that dietary supplementation with isochlorogenic acid significantly increased ileal secretory immunoglobulin A levels and serum immunoglobulin G concentrations in broilers, indicating improved immune function. SICA significantly improves the immune performance, egg quality, and lipid metabolism of laying hens during egg production [8]. In addition, supplementation with SICA enhances immune responses, including immunoglobulin M (IgM), immunoglobulin G (IgG), immunoglobulin A (IgA), complement 3 (C3), complement 4 (C4), and tumor necrosis factor-α (TNF-α) in broilers, and further ameliorates feed digestibility, intestinal morphology, and antioxidant capacity [9].

Proteomic techniques based on liquid chromatography–mass spectrometry have been used to identify and analyze differentially expressed proteins (DEPs) to elucidate the molecular mechanisms underlying immunity [12]. Dietary supplementation with SICA improves the growth performance and immune function of broilers [10]. However, its underlying mechanisms remain underexplored. Therefore, this study established a cyclophosphamide-induced immunosuppressive broiler model to elucidate the peripheral immune regulatory mechanism of SICA in the spleen using proteomics. We evaluated whether SICA ameliorates cyclophosphamide-induced immunosuppression in broilers. Furthermore, we sought to uncover the pivotal metabolic pathways and protein networks involved in this immunomodulatory process, beyond phenotypic observations, to provide mechanistic insights. To provide robust validation of the proteomic findings and establish a reliable mechanistic basis, the identified key DEPs were verified using quantitative polymerase chain reaction (qPCR) analysis and molecular docking studies.

## 2. Materials and Methods

### 2.1. SICA, Animals, and Dietary Treatments

All experimental procedures were approved by the Research Ethics Committee of Hebei Agricultural University (2022143). SICA was provided by Chenguang Biotechnology Group Co., Ltd. (Hebei, China; batch number 2-0969-220217). It contained isochlorogenic acid ≥ 50.0% (isochlorogenic acid A ≥ 12%, isochlorogenic acid B ≤ 15%, and isochlorogenic acid C ≥ 20%), 7~10% other caffeoylquinic acids, 4~5% chlorogenic acid derivatives, 4~5% flavonoids, 9~10% glycosides, 14.0 ± 1.5% ash, and 3~7% moisture. Animal and dietary treatments were performed according to the methods described in a previous study [13] with slight modifications. A total of 432 one-day-old healthy Ross 308 broilers, whose average weight was 48.0 ± 2.0 g, were purchased and raised on Boye Broiler Farm in Baoding City, Hebei Province. The indoor temperature was controlled at 32 °C~34 °C during the 1st to 7th day, then steadily lowered by 3 °C every week until it was reduced to 22 °C~24 °C. The lighting regimen consisted of 23 h of white light exposure followed by 1 h of darkness per day, and broilers were allowed ad libitum access to tap water. The composition of the basal diet is shown in Table 1.

The broilers were raised in two-tiered stainless-steel cages, with each tier having a unified size (length 1.2 m × width 1.2 m × height 0.8 m). Specifically, 12 broilers from each replicate were housed in the first tier to maintain appropriate temperature during the initial period (1~21 d), after which they were redistributed, and 6 of the 12 broilers were randomly assigned to the second tier to reduce stocking density during the growth period (22~42 d). The standard immunization program for broilers was followed, including vaccination against Newcastle disease, avian influenza (H9 subtype), and infectious bronchitis bivalent inactivated vaccine (La Sota strain + H120 strain) on day 7 [14].

### 2.2. Animal Groups and Sample Collection

The animal groups and SICA supplementation regimen were based on the isochlorogenic acid experimental design for laying hens published by Wen et al. [7], with minor modifications. Broilers were randomly assigned to 6 groups (each with 6 replicates of 12 broilers) using SPSS 23.0. This was achieved by generating a random number for each broiler using the RV.UNIFORM function, sorting the list accordingly, and then assigning the birds to the groups sequentially. The treatments were as follows: (1) Control group receiving the basal diet and an intraperitoneal injection of sterile saline (CON); (2) Model group receiving the basal diet and an intraperitoneal injection of cyclophosphamide (MOD); (3) SICA groups receiving the basal diet supplemented with a low dose of 100 mg/kg (L-SICA), a moderate dose of 200 mg/kg (M-SICA) and a high dose of 400 mg/kg of SICA (H-SICA), each followed by intraperitoneal cyclophosphamide; and (4) Antibiotic group receiving the basal diet supplemented with 50 mg/kg aureocycin and intraperitoneal cyclophosphamide (ANT). Based on the method described in a previous report in which immunosuppression was induced by intraperitoneal injection of 80 mg/kg cyclophosphamide [15], we conducted a preliminary experiment to determine the appropriate cyclophosphamide and SICA doses prior to the formal trial. Thus, 80 mg/kg cyclophosphamide was administered to the MOD, L-SICA, M-SICA, H-SICA, and ANT groups, and an equal volume of sterile saline was administered to the CON group on days 37, 38, and 39.

At the end of the experiment (day 42), following a 12 h fasting period that began at 20:00 on day 41, 6 broilers were randomly selected from each group. Blood was collected from the wing vein, centrifuged at 3000× *g* for 10 min at 4 °C, and serum was harvested for subsequent assays. Broilers were euthanized by cervical dislocation for tissue collection. The spleen, thymus, and bursa of Fabricius were separated out, carefully cleared of connective tissue, rinsed with physiological saline, and gently blotted dry on filter paper to remove surface blood. Immune organs were weighed to calculate immune organ indices, after which one-third of each spleen was fixed in 10% formaldehyde, and the remaining tissue was stored at −80 °C for further analyses.

### 2.3. Determination of Immune Organ Indices

Immune organ indices, including those of the spleen, thymus, and bursa of Fabricius, were calculated according to the following formulas:

Immune organ index (mg/g) = immune organ weight (mg)/body weight (g).

### 2.4. Determination of Histological Examination

Hematoxylin–eosin (HE) staining was used for examining the histopathology of the spleen tissue according to a previous study [16] with slight modifications. First, the spleen tissue was fixed in 10% formaldehyde and dehydrated in ethanol. The tissue was embedded in paraffin, and sections were prepared and mounted on glass slides. Sections were deparaffinized in xylene, hydrated through graded ethanol, and rinsed with distilled water. Images were captured randomly at 200× and 400× magnification using a light microscope. The integrity of splenic tissue architecture, including white pulp, red pulp, and lymphocytes, was examined and analyzed.

### 2.5. Determination of Serum Biochemistry

Serum biochemical indicators were measured using an enzyme-linked immunosorbent assay (ELISA) following a previously described method [17]. Serum levels of pro-inflammatory cytokines, including interleukin-2 (IL-2), interferon-γ (IFN-γ), interleukin-1β (IL-1β), and TNF-α, and the anti-inflammatory cytokine interleukin-4 (IL-4), as well as immunoglobulins IgA, IgM, and IgG and complement components C3 and C4, were determined strictly according to the manufacturers’ instructions.

### 2.6. Preparation of Protein and Peptide Samples for iTRAQ-Based Proteomics Analysis

Protein extraction and quantification were performed according to a previously described method with modifications [18]. Frozen spleen tissue (5 mg) was ground in liquid nitrogen, followed by the addition of 50 μL protein lysis buffer (Saiwen Innovation Biotechnology Co., Ltd., Beijing, China) containing 8 M urea, 1% sodium dodecyl sulfate, and a protease inhibitor cocktail. Samples were thoroughly mixed and lysed on ice for 30 min with vortexing every 5 min for 5~10 s. Tissue homogenates were centrifuged at 16,000× *g* for 30 min at 4 °C, and supernatants were collected. Protein concentration was measured using a bicinchoninic acid assay kit, and samples were adjusted to an appropriate concentration. The protein was subjected to sodium dodecyl sulfate-polyacrylamide gel electrophoresis (SDS-PAGE) to detect the quality of the protein for subsequent experiments.

50 μg protein in 45 μL of 8 M protein lysis buffer was mixed with 5 μL 1 M triethylammonium bicarbonate (TEAB) and 1 μL 0.5 M Tris(2-carboxyethyl) phosphine, followed by incubation at 37 °C for 1 h. Iodoacetamide (2 μL, 1 M) was then added for further reaction at 25 °C for 40 min in the dark. After centrifugation at 10,000× *g* for 20 min, the precipitate was dissolved in 100 μL 100 mM TEAB. Finally, 0.625 μL 0.25% trypsin (2 μg/μL) was added at a 1:50 (enzyme/substrate, m/m) ratio for overnight hydrolysis at 37 °C to obtain peptide samples [19].

### 2.7. DIA Mass Spectrometry Detection

Equal amounts of peptide samples were collected to extract peptide segments in a vacuum centrifuge after trypsin digestion. Subsequently, 0.1% trifluoroacetic acid was used to dissolve peptide segments. The hydrophile-lipophile balance was used to desalinate the peptide segments, and the vacuum concentrator was used for drying. Finally, a peptide quantification kit (item number: 23275; Thermo Fisher Scientific, Waltham, MA, USA) was used for peptide quantification in subsequent onboard testing [20].

Detection parameters were as follows: chromatography was performed using Vanquish Neo (Thermo Fisher Scientific, Waltham, MA, USA) and mass spectrometry using an Astral instrument (Thermo Fisher Scientific, Waltham, MA, USA). Chromatographic separation time was 8 min. Buffer A contained 2% acetonitrile and 0.1% formic acid, and buffer B contained 80% acetonitrile and 0.1% formic acid. Positive ion mode was used with a source voltage of 1.5 kV. Both MS and MS/MS data were collected. The analysis column was a uPAC High Throughput column (75 μm × 5.5 cm, Thermo Fisher Scientific, Waltham, MA, USA). Data acquisition was performed using Thermo Xcalibur 4.7 (Thermo Fisher Scientific, Waltham, MA, USA) in DIA mode.

### 2.8. Protein Identification and Bioinformatics Analysis

Mass spectrometry data were processed using Mascot 2.2 and Proteome Discoverer 1.4. The above adjustment factor was changed by more than 1.2-fold, or the down adjustment factor was less than 0.83 times, and *p* < 0.05 was used to screen differentially expressed proteins (DEPs) [21]. Protein changes were visualized using a volcano plot. Principal component analysis (PCA) was conducted to assess intra- and inter-group clustering patterns. DEPs were subjected to a hierarchical clustering algorithm, followed by a correlation analysis, and representation of the data in the form of heat maps. GO enrichment analysis was performed using BLAST2GO (Basic 6.0), and Kyoto Encyclopedia of Genes and Genomes (KEGG) pathway annotation enrichment analysis was performed using the KEGG automatic annotation server (KAAS) software (Version 2.1) [22].

### 2.9. Quantitative PCR Analysis

Quantitative PCR was performed according to previously described methods with minor modifications [23,24]. Total RNA was extracted from spleen tissue using an RNA Easy Fast animal tissue extraction kit (Tiangen Biotech Co., Ltd., Beijing, China), and purity was assessed by the 260/280 absorbance ratio using UV spectrophotometry (UV-2100 model, UNICO Instruments Co., Ltd., Shanghai, China). cDNA was synthesized using a FastKing gDNA Dispelling RT SuperMix kit (Tiangen Biotech Co., Ltd., Beijing, China). Each 20 μL reaction contained 4 μL 5 × FastKing-RT SuperMix, 12 μL total RNA, and 4 μL RNase-free water. Reverse transcription was carried out at 42 °C for 15 min and 95 °C for 3 min. qPCR was conducted using SYBR Green I chemistry, specific primers (Tiangen Biotech Co., Ltd., Beijing, China), and cDNA template. Each 20 μL qPCR reaction contained 10 μL 2 × Fast Real qPCR PreMix (SYBR Green), 0.6 μL forward primer (10 μM), 0.6 μL of reverse primer (10 μM), 2 μL of 50 × carboxy-x-rhodamine reference dye, 2 μL of synthesized cDNA, and 4.8 μL of RNase-free water. The cycling conditions were as follows: 95 °C for 2 min, followed by 40 cycles of 95 °C for 5 s, 55 °C for 10 s, and 72 °C for 15 s. The 2^−ΔΔCt^ method was used for analysis, and glyceraldehyde-3-phosphate dehydrogenase (*GAPDH*) was used as the standard internal reference gene to calculate the relative mRNA level. Primer sequences for the screened differential proteins are shown in Table 2; primers were synthesized based on General Biology.

### 2.10. Molecular Docking Analysis

Molecular docking analysis was performed according to a method described in a previous study [25] with slight modifications. First, the chemical structure of SICA was downloaded from the PubChem database as a docking ligand. Second, the three-dimensional structures of the DEPs related to immune regulation, selected from the PDB database as docking receptors, were downloaded. Third, the water molecules from the receptor were removed and hydrogenated using the AutoDock molecular docking software (Version 4.26), and the possible docking positions between SICA and immunomodulatory differential proteins were plotted. The PyMOL module (Version 3.1) was used for visualization and drawing [26]. Finally, further calculations of molecular dynamics and binding free energy were performed to reveal the binding sites and possible mechanisms of SICA with immunomodulatory DEPs.

### 2.11. Statistical Analysis

All data were tested for normality (Shapiro–Wilk test) and homogeneity of variances (Levene’s test) prior to analysis. Once the data satisfied the assumptions for the parametric tests (*p* > 0.05 for both tests), they were expressed as the mean ± standard error of the mean and analyzed using one-way analysis of variance (ANOVA). Statistical analysis was performed using SPSS 23.0, and significant differences were determined using Duncan’s test at *p* < 0.05.

## 3. Results

### 3.1. Effect of SICA on the Immune Organ Indices

**Figure 1:**
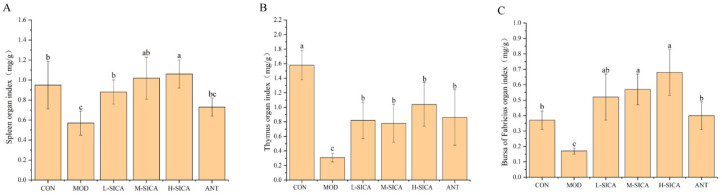
The effect of SICA on the immune organ indices (*n* = 6). (**A**) Spleen organ index, (**B**) Thymus organ index, and (**C**) Bursa of Fabricius organ index. Different lowercase letters (a, b, or c) marked on the error lines indicate significant differences (*p* < 0.05).

The immune organ indices are shown in Figure 1. Compared to CON mice, the indices for the spleen, thymus, and bursa of Fabricius were significantly reduced in MOD mice on day 42 (*p* < 0.05). Compared to MOD, the indices of the spleen, thymus, and bursa of Fabricius were significantly increased in the SICA treatment groups and ANT on day 42 (*p* < 0.05). Therefore, SICA treatment increased the immune organ indices of broilers, indicating its potential to improve immune function.

### 3.2. Effect of SICA on the Spleen Histological Examination

**Figure 2:**
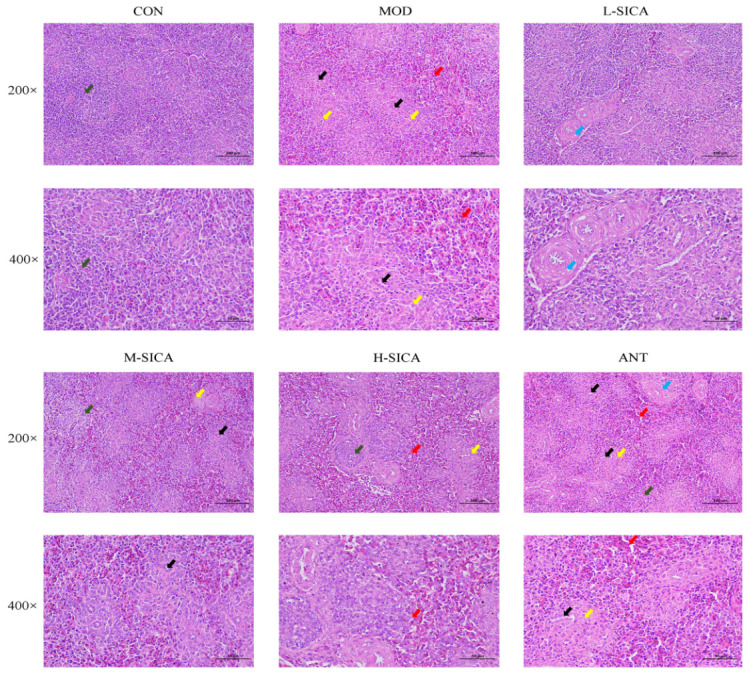
The impact of SICA on the spleen, as demonstrated by histological examination. 200× and 400× in the figure represent the total magnification of the eyepiece and objective lens of the optical microscope. Note: Presence of the lymph nodes (green arrows); Marked congestion of the red pulp (red arrows); Proliferation of small vessels (black arrows); Increased perivascular connective tissue (yellow arrows); Cytoplasmic vacuolization in smooth muscle cells of the vessel wall (blue arrows).

Histological examination of the spleen is shown in Figure 2. The CON group displayed a normal structure of the spleen featuring a clear boundary and a reasonable ratio of red and white pulp, with an abundance of lymphocytes. Compared to the CON group, the spleens of the MOD group showed significant abnormalities: the white pulp area was reduced, and red pulp congestion was visible. The number of lymphocytes decreased, and small vessels proliferated. These results indicated that the spleen was severely damaged, and an immunosuppressive model was successfully established.

Notably, compared with the MOD group, the histological examination of the spleen in the SICA treatment group showed recovery, with reduced damage to the white pulp area and congestion of the red pulp, a clearer boundary between the white pulp and red pulp, and a tendency towards development of a normal architecture. The number of lymphocytes and the amount of connective tissue significantly increased, and small blood vessel proliferation was observed in a small area. The M-SICA and H-SICA groups showed significantly better results than the L-SICA group. In addition, compared to ANT, SICA treatment showed better improvement in the injured spleen.

### 3.3. Effect of SICA on the Serum Biochemistry

**Figure 3:**
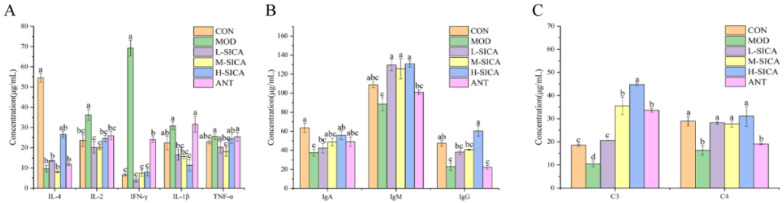
The effect of SICA on immune factors (*n* = 6). (**A**) The expression levels of inflammatory factors (IL-4, IL-2, IFN-γ, IL-1β, and TNF-α), (**B**) The expression level of immunoglobulins (IgA, IgM, and IgG), and (**C**) The expression levels of complement (C3 and C4 contents). Different lowercase letters (a, b, c, or d) were marked on the error lines indicating significant differences (*p* < 0.05).

Serum biochemical results are shown in Figure 3. Compared with the CON group, the anti-inflammatory factor IL-4 content in the MOD group significantly decreased (*p* < 0.05). However, in broilers receiving the H-SICA treatment, IL-4 levels significantly increased relative to those of the MOD group (*p* < 0.05). Compared with the CON group, the pro-inflammatory factors IL-2, IFN-γ, and IL-1β were significantly elevated (*p* < 0.05) in the MOD group. In contrast, relative to the MOD group, the L-SICA, M-SICA, and H-SICA treatment groups showed significant reductions in IL-2, IFN-γ, and IL-1β contents (*p* < 0.05). For TNF-α, no significant difference between the CON and MOD groups; however, relative to the MOD group, TNF-α significantly decreased (*p* < 0.05) in the M-SICA group. Unlike the SICA-treated groups, the ANT group showed no significant differences in IL-4, IL-1β, or TNF-α compared with the MOD group.

Compared with the CON group, there were significant reductions in immunoglobulin levels, including IgA, IgM, and IgG (*p* < 0.05), in the MOD group. As expected, SICA treatment reversed this phenomenon. Specifically, compared with the MOD group, both IgA and IgG contents significantly increased in the H-SICA group (*p* < 0.05), whereas IgM levels significantly increased in the L-SICA, M-SICA, and H-SICA groups (*p* < 0.05). However, in the ANT group, immunoglobulin contents did not differ significantly from those in the MOD group.

Compared with the CON group, complement components C3 and C4 were significantly decreased in the MOD group (*p* < 0.05). Relative to the MOD group, the C3 content significantly increased in the L-SICA, M-SICA, H-SICA, and ANT groups (*p* < 0.05), whereas the C4 content significantly increased in the L-SICA, M-SICA, and H-SICA groups (*p* < 0.05).

### 3.4. Identification of Protein by iTRAQ-Based Proteomics Analysis

**Figure 4:**
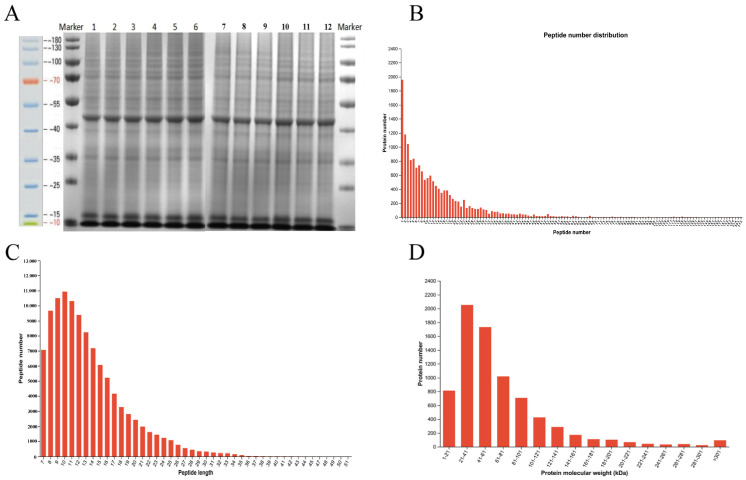
Basic information for iTRAQ identification of peptides and proteins. (**A**) SDS-PAGE image of spleen proteins, (**B**) Distribution of protein identifications by peptide number, (**C**) Distribution of peptide lengths, and (**D**) Distribution of protein molecular weights.

Based on the above-mentioned results, the dietary supplement of 400 mg/kg SICA was selected for proteomic determination and subsequent validation experiments. Basic information for the isobaric tags for relative and absolute quantitation (iTRAQ) identification of peptides and proteins is shown in Figure 4. Spleen proteins were intact and displayed consistent bands without tailing (Figure 4A). A total of 1959 proteins were identified with one peptide, whereas 11,556 proteins were identified with 2~23 peptides (Figure 4B). The distribution of peptide lengths was mainly between 7 and 20 amino acids, indicating that the test data were of high quality (Figure 4C). Furthermore, more than 81% of peptides were <100 kDa (Figure 4D), meeting the quality control requirements. A total of 7723 protein groups (clusters of proteins sharing an identical peptide set) were identified through data processing of the iTRAQ-derived information, including 885 protein groups with a single unique peptide and 6838 with two or more peptides. All subsequent analyses were based on these protein groups.

**Figure 5:**
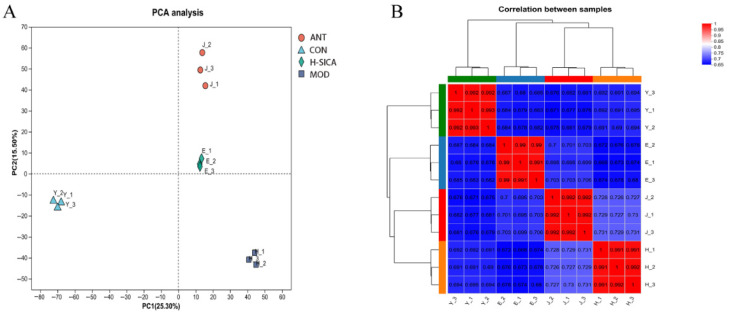
The protein principal component analysis and correlations. (**A**) The protein PCA and (**B**) Protein correlation analysis.

The PCA and correlation results are shown in Figure 5. The PCA indicated that samples within each treatment group clustered closely together, demonstrating satisfactory reproducibility. Samples from different treatment groups were clearly separated, showing distinct inter-group differences and strong treatment specificity among the CON, MOD, H-SICA, and ANT groups (Figure 5A). To further evaluate experimental reliability, correlations of protein expression levels between samples were calculated using Pearson’s correlation coefficient (r). As shown in Figure 5B, red grids indicate strong intra-group correlations (r > 0.99), whereas blue grids reflect weak inter-group correlations, confirming high intra-group similarity and significant inter-group differences among the CON, MOD, H-SICA, and ANT groups.

### 3.5. Identification of DEPs

**Figure 6:**
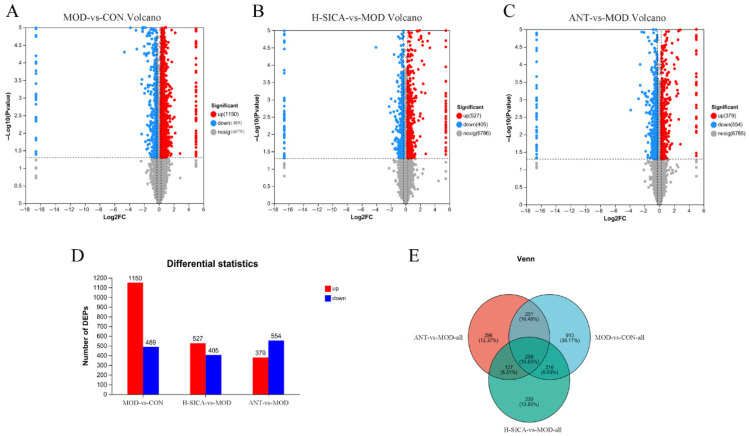
Inter-group DEPs volcano plots of the spleen tissue. (**A**) The DEP volcano diagram of the MOD group compared with the CON group, (**B**) The DEP volcano diagram of the H-SICA group compared with the MOD group, (**C**) The DEP volcano diagram of the ANT group compared with the MOD group, (**D**) The statistical histogram of inter-group DEPs, and (**E**) The Venn diagram of inter-group DEPs.

Figure 6 shows the identification of DEPs. The abscissa indicated the fold change values (log2 transformed), and the ordinate corresponded to the *p*-value (−log10 transformed). A total of 1639 differential proteins were identified between the MOD and CON groups, with 1150 upregulated and 489 downregulated. Relative to the MOD group, 932 DEPs were identified in the H-SICA group, including 527 upregulated and 405 downregulated proteins. Between the ANT and MOD groups, 933 DEPs were identified, of which 379 were upregulated, and 554 were downregulated.

### 3.6. GO Enrichment Classification of DEPs

**Figure 7:**
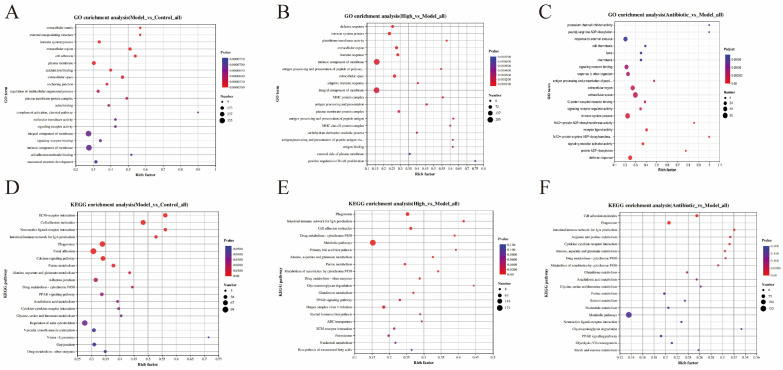
GO and KEGG enrichment analysis. (**A**) The GO enrichment results of MOD compared with CON, (**B**) The GO enrichment results of H-SICA compared with MOD, (**C**) The GO enrichment results of ANT compared with MOD, (**D**) The KEGG enrichment results of MOD compared with CON, (**E**) The KEGG enrichment results of H-SICA compared with MOD, and (**F**) The KEGG enrichment results of ANT compared with MOD.

Enrichment results under “biological process,” “cellular component,” and “molecular function” domains were ordered by *p*-value, and the top 20 terms with the smallest *p*-values were summarized. Figure 7 shows enrichment results for each group. In Figure 7A, seven pathways—“integral component of membrane,” “intrinsic component of membrane,” “immune system process,” “extracellular region,” “cell adhesion,” “plasma membrane,” and “anatomical structure development”—were significantly affected in broilers administered cyclophosphamide. In Figure 7B, seven pathways, “integral component of membrane,” “intrinsic component of membrane,” “immune system process,” “extracellular region,” “extracellular space,” “immune response,” and “defense response,” were significantly influenced in immunosuppressed broilers receiving H-SICA treatment. In Figure 7C, nine pathways, “extracellular region,” “extracellular space,” “immune system process,” “defense response,” “signaling receptor activator activity,” “receptor ligand activity,” “signaling receptor binding,” “response to external stimulus,” and “response to other organism” were significantly affected in broilers receiving ANT treatment. In summary, the common enriched pathways across groups were “extracellular region” and “immune system process.”

### 3.7. KEGG Enrichment Classification of DEPs

KEGG analysis was performed using the KOBAS database, and the results were visualized using a scatter plot. The screened KEGG pathways were designated as those with *p* < 0.05. Figure 7 shows the KEGG pathway enrichment levels. Figure 7D showed that nine pathways, “extracellular matrix (ECM) receptor interaction,” “phagosome,” “cell adhesion molecules,” “focal adhesion,” “calcium signaling pathway,” “regulation of actin cytoskeleton,” “purine metabolism,” “intestinal immune network for IgA production,” and “peroxisome proliferator-activated receptor (PPAR) signaling pathway,” had significant effects in the MOD group compared with the CON group. Figure 7E showed that ten pathways, “phagosome,” “cell adhesion molecules,” “metabolic pathways,” “purine metabolism,” “alanine, aspartate and glutamate metabolism,” “herpes simplex virus 1 infection,” “PPAR signaling pathway,” “ECM receptor interaction,” “intestinal immune network for IgA production,” and “biosynthesis of unsaturated fatty acids” had significant effects in the H-SICA group compared with that recorded in the MOD group. Figure 7F showed that seven pathways, “metabolic pathways,” “phagosomes,” “cell adhesion molecules,” “PPAR signaling pathway,” “purine metabolism,” “alanine, aspartate and glutamate metabolism,” and “intestinal immune network for IgA production,” had significant effects in the ANT group compared with those in the MOD group. In summary, the commonly enriched immune-related pathways among the CON, MOD, H-SICA, and ANT groups were mainly “cell adhesion molecules,” “phagosomes,” and “intestinal immune network for IgA production.”

### 3.8. Selection of DEPs

**Figure 8:**
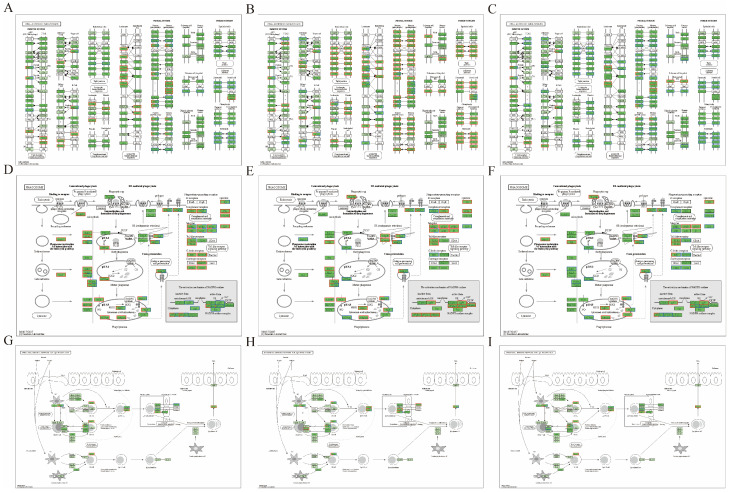
Protein expression results. (**A**) The protein expression result of MOD compared with CON in the cell adhesion molecule pathway, (**B**) The protein expression result of H-SICA compared with MOD in the cell adhesion molecule pathway, (**C**) The protein expression result of ANT compared with MOD in the cell adhesion molecule pathway, (**D**) The protein expression result of MOD compared with CON in the phagocytic pathway, (**E**) The protein expression result of H-SICA compared with MOD in the phagocytic pathway, (**F**) The protein expression result of ANT compared with MOD in the phagocytic pathway, (**G**) The protein expression result of MOD compared with CON in the intestinal immune network for IgA production pathway, (**H**) The protein expression result of H-SICA compared with MOD in the intestinal immune network for IgA production pathway, and (**I**) The protein expression result of ANT compared with MOD in the intestinal immune network for IgA production pathway.

DEPs were defined based on the above three immune pathways and are shown in Tables S1–S3 and Figure 8. *PTPRC*, an important positive regulatory factor in T- and B-cell antigen receptor signaling, was identified in the “cell adhesion molecule” pathway. Compared with the CON group, the expression level of *PTPRC* was downregulated in the MOD group, whereas compared with the MOD group, *PTPRC* expression was upregulated in the H-SICA and ANT groups. p67phox, a factor involved in preventing excessive expression of apoptotic genes and reducing viral inflammation, was identified in the “phagosome” pathway. Compared with the CON group, the expression level of p67phox was downregulated in the MOD group, whereas compared with the MOD group, p67phox expression was upregulated in the H-SICA and ANT groups. *BCR*, a B-cell antigen receptor involved in regulating abnormal adhesion of hematopoietic cells; MHCII, a molecule expressed by specialized antigen-presenting cells; and *CCR9*, a chemokine receptor that activates T lymphocytes, were identified in the “intestinal immune network for IgA production” pathway.

### 3.9. Effect of SICA on mRNA Expression of DEPs

**Figure 9:**
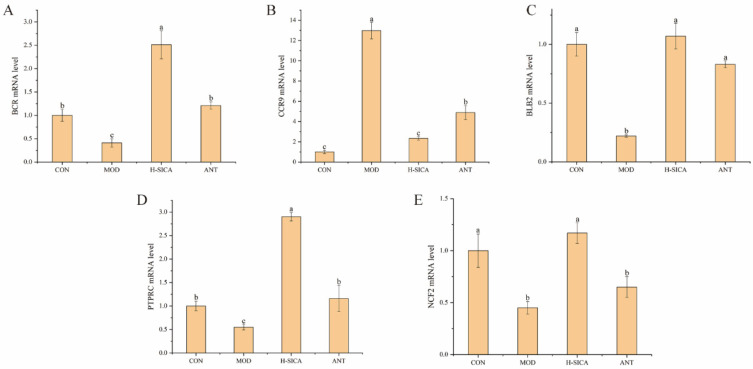
Differential expression of genes corresponding to DEPs at the mRNA level. (**A**) *BCR*, (**B**) *CCR9*, (**C**) *BLB2*, (**D**) *PTPRC*, and (**E**) *NCF2*. Different lowercase letters (a, b, or c) above the error bars indicate significant differences (*p* < 0.05).

qPCR was performed to determine mRNA transcript levels of the five screened DEPs (Figure 9). Compared with the CON group, the expression levels of *PTPRC*, *NCF2*, *BCR*, and *BLB2* in the spleens of MOD mice were significantly downregulated (*p* < 0.05). Conversely, compared with the MOD group, the expression levels of *PTPRC*, *NCF2*, *BCR*, and *BLB2* were significantly upregulated in the H-SICA group (*p* < 0.05), and the expression levels of *PTPRC*, *BCR*, and *BLB2* were significantly upregulated in the ANT group (*p* < 0.05). The expression level of *CCR9* was significantly upregulated in the MOD group compared with the CON group (*p* < 0.05), whereas compared with the MOD group, *CCR9* expression was significantly downregulated in both the H-SICA and ANT groups (*p* < 0.05). These results showed that the genes corresponding to the DEPs exhibited marked differential expression at the mRNA level after SICA treatment, indicating that SICA may participate in immune responses, and the above findings were consistent with the proteomics results.

### 3.10. Molecular Docking Studies of SICA and DEPs

**Figure 10:**
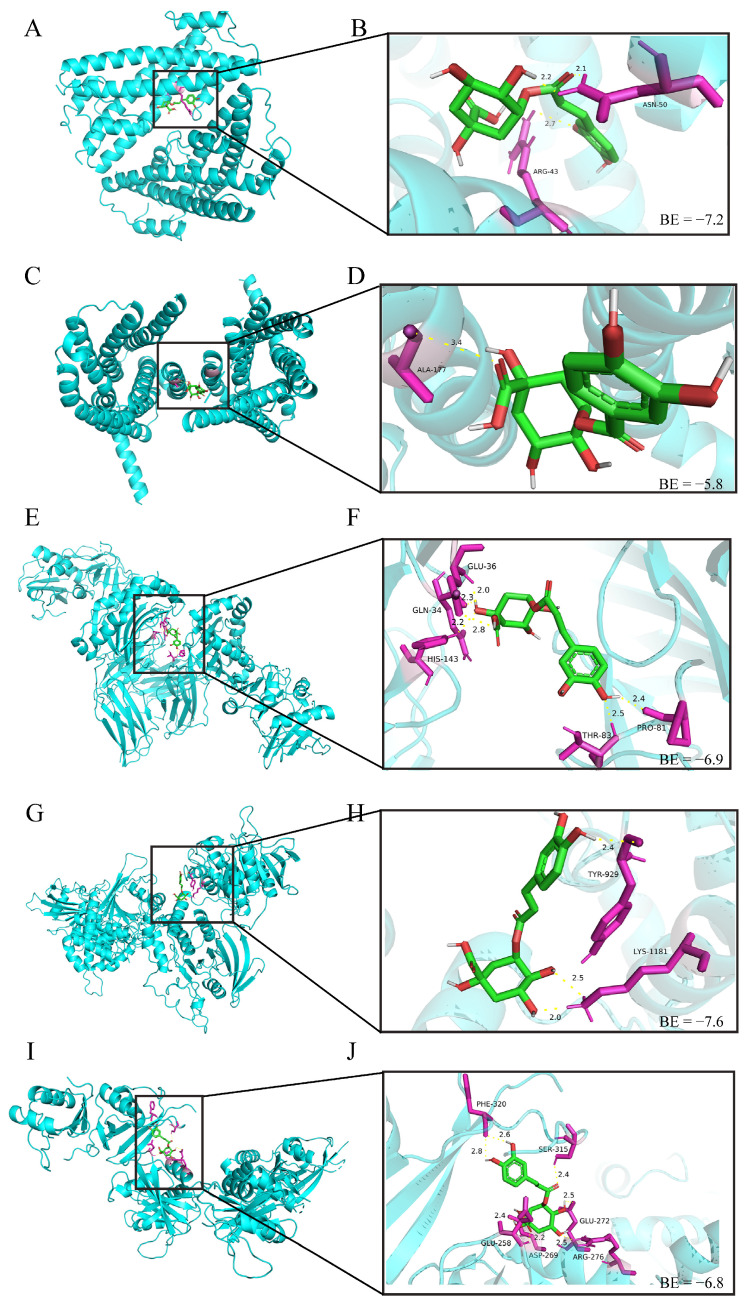
Molecular docking simulation diagram of SICA and DEPs. (**A**,**B**) *BCR* protein, (**C**,**D**) *CCR9* protein, (**E**,**F**) MHCII protein, (**G**,**H**) *PTPRC* protein, and (**I**,**J**) p67phox protein. Binding energy (kcal/mol) values between SICA and DEPs are indicated.

Molecular docking was performed to simulate interactions between SICA and the identified DEPs. The binding energy was used to evaluate the binding force between components and their targets. When the binding energy is less than zero, the component can freely bind to the target, and a lower binding energy indicates stronger binding affinity. The docking results for SICA with *BCR*, *CCR9*, MHCII, *PTPRC*, and p67phox are shown in Figure 10. When the binding energy was less than −5 kcal/mol, strong binding capacity was observed. Among the targets, p67phox exhibited the strongest binding affinity with SICA.

## 4. Discussion

Antibiotic-free farming plays an important role in the development of the livestock and poultry industries and has promising prospects. Natural active substances have great potential as alternatives to antibiotics. Immune organs represent specialized organs in animals that are primarily responsible for immune defense and directly reflect immune function [27]. Thus, increases in immune organ indices indicate enhanced immune activity. In this study, SICA treatments significantly increased the spleen, thymus, and bursa of Fabricius indices (*p* < 0.05), which is consistent with previous findings showing that *Lonicerae flos* chlorogenic acid improved immune organ indices in broilers, including liver and spleen indices [28].

The spleen contains numerous lymphocytes, functions as a blood reservoir, and plays crucial roles in immune regulation [29]. As the largest peripheral lymphoid organ, it undergoes excessive apoptosis and stress responses following cyclophosphamide stimulation, resulting in inhibition of anti-inflammatory cytokines, overexpression of inflammatory cytokines, and subsequent suppression of immune function [30]. Our results indicated that SICA significantly ameliorated cyclophosphamide-induced splenic damage in broilers by reducing white and red pulp lesions and increasing lymphocytes and connective tissue. These findings are consistent with a previous report demonstrating that chlorogenic acid from *Lysimachia christinae* reduced triptolide-induced impairments in spleen, liver, kidney, and heart function [31].

Inflammatory factors, immunoglobulins, and complements in serum participate in humoral immunity and reflect the strength of immune responses [32]. Immunomodulatory effects of chlorogenic acid–related compounds have been reported in several animal models. For example, Zhou et al. [12] observed a linear increase in serum complement C3 (*p* = 0.004) and a quadratic increase in serum IgG (*p* = 0.005) in Cobb broilers after 42 days of dietary supplementation with 500~3000 mg/kg isochlorogenic acid. Wen et al. [7] found that dietary supplementation with 50 or 100 mg/kg stevia extract (≥50% isochlorogenic acid) for 24 weeks significantly increased serum IgM and IgG in 46-week-old Roman laying hens (*p* < 0.05). The results of this study differ from ours in several key respects. First, laying hens have a longer growth cycle, which allows their immune system adequate time to develop and mature. They maintain immune homeostasis over an extended period to cope with the demands of egg production and exhibit greater tolerance to acute stress, which may explain the lower dosage used in their case [33]. In contrast, broilers have a short and rapid growth cycle with a relatively underdeveloped immune system. The Ross broilers used in our study were specifically characterized by fast growth and high stress sensitivity. Furthermore, isochlorogenic acid was supplemented in the diet of laying hens without an immunosuppression model in Wen’s study, whereas our study employed an immunosuppression model induced by cyclophosphamide in broilers. These factors may collectively explain the differences observed between the two studies. Shang et al. [34] reported that supplementation with 400~800 mg/kg chlorogenic acid significantly increased serum C3 and C4 concentrations (*p* < 0.05) and enhanced immune function in common carp. The distinct structural features of SICA, including the caffeoyl and quinic acid moieties, catechol groups, and unsaturated double bonds, contribute to strong antioxidant and immunomodulatory activities [12]. However, the immunomodulatory properties of SICA vary depending on dosage, species, and experimental model. In this study, supplementation with 400 mg/kg of SICA significantly modulated serum immune markers by decreasing pro-inflammatory cytokines and enhancing anti-inflammatory cytokines, immunoglobulins, and complements.

Proteomics, a concept first proposed by Wilkins and Willian in 1994 [35], refers to the complete set of proteins expressed by a genome [36]. Isobaric tags for relative and absolute quantitation (iTRAQ) provide a relative and absolute quantitative technique for in vitro isotopic labeling [37]. This method rapidly and comprehensively analyzes protein expression changes in target cells, tissues, and organs; identifies specific differentially expressed proteins; explains biological processes; and elucidates molecular mechanisms [38,39]. Therefore, iTRAQ-based proteomics plays a crucial role in evaluating immune mechanisms [40]. However, proteomic studies investigating the immunomodulatory mechanisms of bioactive substances in animals remain limited. Current research has identified diverse pathways, such as those related to oxidative stress or apoptotic signaling; however, no reports directly aligning with our unique pathway have been identified to date. For example, proteomic analysis by Zhao et al. revealed that the immune-restorative effects of *Cistanche deserticola* polysaccharides in mouse thymus were linked to upregulation of proteins in the T-cell receptor signaling pathway, suggesting enhanced T-cell signaling as a core mechanism. This T-cell activation can be further amplified by cytokines such as IFN-γ, which is derived from Th1 cells and NK cells. IFN-γ enhances antigen recognition by upregulating MHC-I/II expression and promotes T-cell homing through the secretion of chemokines like CXCL10 [41]. He et al. reported that 149 DEPs were upregulated in macrophages following egg white glycopeptide treatment, mapping to 76 KEGG pathways (e.g., “NF-kappa B signaling pathway,” “TNF signaling pathway,” “C-type lectin receptor signaling pathway”) and 12 protein domains (e.g., “Lectin C-type domain,” “Immunoglobulin domain,” “Immunoglobulin V-set domain”). Thus, his proteomics study led him to conclude that egg white glycopeptide can influence macrophage function through the regulation of multiple protein domains, demonstrating its immunomodulatory effects [38].

In our study, SICA modulated immune responses through pathways involving cell adhesion molecules, phagosomes, and the intestinal immune network for IgA production. SICA upregulated *BCR*, MHCII, *PTPRC*, and p67phox, and downregulated *CCR9*. *BCR*, p67phox, and MHC II form a precise “recognition-regulation-presentation” immunoregulatory circuit. Upon antigen recognition, *BCR* not only facilitates the loading of idiotype peptides derived from its variable region onto MHCII molecules for presentation to CD4^+^ T cells—thereby initiating B-cell/T-cell collaboration—but also coalesces with MHCII in membrane microdomains such as lipid rafts, which enhances tyrosine phosphorylation and further amplifies *BCR* signaling [42,43]. p67phox, a key subunit of NADPH oxidase, serves as the central regulator of reactive oxygen species (ROS) generation in this process. Dysfunction of p67phox leading to ROS imbalance directly impairs the effective initiation of *BCR* signaling, thereby reducing B-cell activation efficiency and the strength of the humoral immune response. Concurrently, p67phox acts as a modulator of MHCII function by fine-tuning ROS levels to ensure efficient antigen presentation and subsequent T-cell responses while preventing excessive oxidative damage. Furthermore, elevated p67phox-mediated ROS may downregulate the *CCR9* receptor both functionally and at the expression level: ROS can oxidatively damage *CCR9*-mediated signal transduction, leading to its functional inactivation, and promote receptor internalization, degradation, and suppression of *CCR9* gene expression, thereby reducing the available receptor pool [44,45,46,47]. Theoretically, this mechanism could interfere with organ-specific metastasis driven by the *CCR9*-CCL25 axis in certain tumor cells. *PTPRC*, a critical protein tyrosine phosphatase, functions as an upstream signaling hub in this network. Its up-regulation enhances the dephosphorylation and activation of Src-family kinases, thereby potently and directly promoting the initiation and amplification of the *BCR* signaling pathway. This in turn indirectly improves the efficiency of MHCII-mediated antigen presentation and *CCR9*-dependent lymphocyte homing. The relationship with p67phox is more indirect and context-dependent, primarily involving the modulation of immune-cell activation states that may potentially influence ROS production [48,49,50]. In summary, proteomic techniques may become powerful tools for targeted evaluation of SICA-mediated immunoregulation in cells and animal models.

It is acknowledged that mRNA expression levels do not always directly correlate with corresponding protein abundance due to post-transcriptional processes (e.g., polyadenylation, capping, and splicing) and translational or post-translational regulation (e.g., modifications, disulfide bond formation, cleavage, and polymerization) [51]. However, given its high sensitivity, suitability for profiling multiple targets, rapid turnaround, and cost-effectiveness, qPCR is commonly employed as a complementary method to provide indirect corroborative evidence for proteomic findings [52]. In this study, primers were designed for the DEPs, and mRNA expression of *BCR*, *CCR9*, MHCII, *PTPRC*, and p67phox was validated through qPCR. The upregulation of *BCR*, *PTPRC*, *BLB2*, and *NCF2*, along with the downregulation of *CCR9*, was consistent with the proteomics results. However, a key limitation of the present study is the lack of direct protein-level validation using techniques such as Western blot or immunohistochemistry. Addressing this will be an important focus in our future work to elucidate the mechanism by which SICA modulates immune responses.

These results provide a valuable direction for future mechanistic studies of SICA’s immunomodulatory activity. Molecular docking, a computational structural prediction technique, is widely used to evaluate ligand–protein interactions. It predicts binding affinity and interaction mechanisms by simulating docking between ligand molecules and target biomolecules [53]. SICA exhibited the strongest binding affinity for p67phox, consistent with the observed upregulation of p67phox protein expression in the H-SICA group. We hypothesize that SICA may enhance the stability of p67phox and potentiate its biological functions through strong binding at key protein sites. Further experimental validation is required to confirm the underlying mechanism and assess its functional efficacy.

## 5. Conclusions

In summary, this study showed that SICA can enhance immune organ indices, serum immune factors, and spleen damage induced by cyclophosphamide in immunosuppressed broilers. Furthermore, SICA can activate three pathways including cell adhesion molecules, phagosomes, and the intestinal immune network for IgA production. Five DEPs were identified based on the results of the proteomic analysis. qPCR results showed that *BCR*, MHCII, *PTPRC*, and p67phox proteins were upregulated, and *CCR9* protein was downregulated to regulate immunity. Therefore, we identified a novel bioactive compound that will contribute to healthier and safer broiler farming practices in the future.

## Figures and Tables

**Table 1:** Basal diet and nutrition.

Component	Content (g/kg)	
	Initial Period	Growth Period
Corn (7.5% CP)	508.7	506.7
Soybean meal (44% CP)	346.5	338.7
Corn gluten meal (60% CP)	50.0	50.0
Cottonseed bioactive peptides (46% CP)	0.0	6.0
Wheat bran (14.8% CP)	20.3	22.6
Soybean oil	26.7	26.7
_DL_-methionine	2.8	2.8
_L_-lysine	4.0	4.0
_L_-threonine	1.3	1.3
Choline chloride	1.2	1.2
Monocalcium phosphate (15% Ca, 22.5% P)	15.5	15.3
Calcium carbonate	16.5	16.0
Sodium chloride	1.0	1.0
Sodium bicarbonate	3.0	3.0
Premix	1.0	1.0
Calculated composition		
Metabolizable energy, kcal/kg	3000.0	3000.0
Crude protein	230.0	233.0
Lysine	14.4	14.4
Methionine	6.95	6.94
Threonine	9.70	9.70
Tryptophan	2.50	2.50
Arginine	14.9	15.0
Isoleucine	11.4	11.2
Leucine	21.7	21.5
Ca	9.6	9.6
Available P	4.8	4.8

Note: The dietary comprised the following ingredients per kilogram: Mg, 120 mg; Fe, 20 mg; Cu, 16 mg; Zn, 110 mg; Se, 0.3 mg; I, 1.3 mg; vitamin A, 12,000 IU; vitamin D3, 5000 IU; vitamin E, 80 IU; vitamin B12, 0.017 mg; vitamin K, 3.2 mg; thiamin, 3.2 mg; riboflavin, 8.6 mg; nicotinic acid, 65 mg; pantothenic acid, 20 mg; biotin, 0.2 mg; folic acid, 2.2 mg. CP is crude protein.

**Table 2:** Primer sequences for differentially expressed genes.

Gene Name	Primer	Sequence (5′-3′)	Amplicon Length (bp)	Temperature	GenBank Accession Number
*GAPDH*	Forward	CCGTCCTCTCTGGCAAAGTC	90	60.39	ENSGALT00010053593.1
	Reverse	CCCTTGAAGTGTCCGTGTGT		60.18	
*BCR*	Forward	CCAGTGGCAAGCTGAAGGTA	109	59.96	ENSGALT00010064499.1
	Reverse	CGCAGTGGTGCATTTGTTCA		59.97	
*CCR9*	Forward	GCACAGTGGGAAATGCCTTG	281	60.04	ENSGALT00010042152.1
	Reverse	TCCGCCTTTGCTTGGAAGAT		59.96	
*BLB2*	Forward	CGGCGTTCTTCTTCTACGGT	58	60.11	ENSGALT00010007045.1
	Reverse	CCTGTCCAGAAACCTCACCC		59.96	
*PTPRC*	Forward	TCCTTCTCAAACTCCGACGC	60	60.04	ENSGALT00010029609.1
	Reverse	CCAGCACTGCAATGAACCAC		60.04	
*NCF2*	Forward	TGTTGTGTGAAACGGTTGGG	249	59.19	XM_040677543.2
	Reverse	GCTAGAAAGCAAGCTTAGCAGA		58.73	

Note: *GAPDH*: glyceraldehyde-3-phosphate dehydrogenase; *BCR*: B-cell receptor; *CCR9*: C-C motif chemokine receptor 9; *BLB2* is a gene associated with major histocompatibility complex class II (MHCII); *PTPRC*: Protein Tyrosine Phosphatase Receptor Type C; *NCF2* is a gene associated with Neutrophil cytosolic factor 2 (p67phox).

## Data Availability

All data in this article are presented in the article.

## Supplementary Materials

The following supporting information can be downloaded at: https://www.mdpi.com/article/10.3390/ani16010025/s1, Table S1: The top 20 proteins significantly regulated by MOD compared with CON; Table S2: The top 20 proteins significantly regulated by H-SICA compared with MOD; Table S3 The top 20 proteins significantly regulated by ANT compared to MOD.
