# Supplementary material for: iTRAQ-Based Proteomics Reveals the Potential Mechanisms Underlying Diet Supplementation with Stevia Isochlorogenic Acid That Alleviates Immunosuppression in Cyclophosphamide-Treated Broilers
Source: Animals (Basel). 2025 Dec 22;16(1):25. doi: 10.3390/ani16010025 (PMC12784939; doi:10.3390/ani16010025)
Supplement: Supplementary file 1 [file animals-16-00025-s001.zip › animals-4048262-supplementary.pdf]

Supplemental file

iTRAQ-based proteomics reveals the potential mechanisms underlying diet supplementation with stevia isochlorogenic acid that alleviates immunosuppression in cyclophosphamide-treated broilers

Jiatong Jin [a], Shuqi Zhao [a], Pengyu Zhao [b], Yushuo Zhang [a], Lifei Wu [b], Liangfu Zhou [a], Yasai Sun [a], Wen Zhao [a], Qian Zhou [a, *]

[a] *College of Food Science and Technology, Hebei Agricultural University, Baoding 071001, PR China.*

[b] *Chen Guang Biotechnology Group Co., Ltd., Handan 057250, PR China.*

* Corresponding author: Qian Zhou, College of Food Science and Technology, Hebei Agricultural University, No. 2596 Lekai South Road, Baoding, 071000, China.

E-mail addresses: zhouqian@hebau.edu.cn. Tel.: +86 0312 7528180.

Author Contributions

All authors participated in the design of the study. Jiatong Jin: Methodology, Data curation, Formal analysis, Writing original draft, Software, Investigation. Shuqi Zhao: Validation, Software, Investigation, Data curation. Pengyu Zhao: Project administration. Yushuo Zhang: Software, Validation. Lifei Wu: Project administration. Liangfu Zhou: Supervision, Methodology, Data curation. Yasai Sun: Supervision, Methodology. Wen Zhao: Conceptualization, Project administration, Funding acquisition. Qian Zhou: Writing review & editing, Supervision, Conceptualization, Funding acquisition.

Notes

The authors declare no competing financial interest.

Table S1 The top 20 proteins significantly regulated by MOD compared with CON

Accession	Gene Name	Fold change	Log2FC	P-value	Regulate	Pathway
A0A090HZ95	TNFSF11	1.462	0.5479	0.0116	up	Intestinal immune network for IgA production
A0A173G7C7	MBL	1.451	0.537	0.06405	up	Phagosome
A0A1D5PLI8	BLB1	0.5459	-0.8733	0.07449	down	Phagosome; Cell adhesion molecules; Intestinal immune network for IgA production
A0A1D5PQP8	ITGA9	1.29	0.3674	0.0004129	up	Cell adhesion molecules; Intestinal immune network for IgA production;
A0A223LWZ9	Bu	0.3539	-1.499	0.0004232	down	Cell adhesion molecules
A0A2H4C5K1	BF	0.3678	-1.443	0.0004863	down	Phagosome; Cell adhesion molecules
A0A3Q2TU19	EPX	0.229	-2.127	1.82E-08	down	Phagosome
A0A3Q2UFV6	COMP	1.893	0.9207	0.0258	up	Phagosome
A0A3Q2UG72	C4	1.744	0.8024	0.05543	up	Phagosome
B5BSJ8	BLB2	0.6917	-0.5318	1.07E-05	down	Phagosome;Cell adhesion molecules;Intestinal immune network for IgA production
A0A8V0YDM1	NCF2	0.8236	-0.28	0.03482	down	Phagosome
A0A3Q3A736	COLEC11	1.73	0.7908	0.03956	up	Phagosome
A0A3Q3A741	PTPRE	0.6016	-0.7331	0.01949	down	Cell adhesion molecules
A0A3Q3A7M3	CD99	1.463	0.5489	0.02033	up	Cell adhesion molecules
A0A3Q3AXX7	GP1BB	0.707	-0.5002	0.3337	down	Cell adhesion molecules
A0A452J7Z2	SDC4	2.53	1.339	0.003384	up	Cell adhesion molecules
A0A8V0X1T8	BCHE	1.533	0.6164	0.00359	up	Cell adhesion molecules
A0A8V0XAR1	DMB2	0.7102	-0.4937	0.01878	down	Phagosome;Cell adhesion molecules;Intestinal immune network for IgA production
A0A8V0XB78	COLEC12	1.814	0.8592	2.55E-05	up	Phagosome
A0A8V0XBG4	FNDC3A	0.4087	-1.291	0.006538	down	Cell adhesion molecules

Table S2 The top 20 proteins significantly regulated by H-SICA compared with MOD

Accession	Gene Name	Fold change	Log2FC	P-value	Regulate	Pathway
A0A3Q2TU19	EPX	3.814	1.931	6.26E-08	up	Phagosome
B5BSD6	BF2	5.05	2.336	1.13E-05	up	Phagosome; Cell adhesion molecules
A0A8V0XF54	ALCAM	0.7221	-0.4697	8.88E-05	down	Cell adhesion molecules
A0A8V1ACG8	ITGA2B	1.334	0.4158	0.0004317	up	Intestinal immune network for IgA production; Phagosome; Cell adhesion molecules
B5BSR6	BLB2	0.7897	-0.3406	0.0006797	down	Phagosome; Cell adhesion molecules; Intestinal immune network for IgA production
A0A1D5PLI8	BLB1	1.404	0.4895	0.0008445	up	Phagosome; Cell adhesion molecules; Intestinal immune network for IgA production
A2N883	VH1	1.732	0.7924	0.001055	up	Phagosome; Intestinal immune network for IgA production
B5BSD5	TAP2	45.48	5.507160349	0.001302	up	Phagosome
Q5ZFP5	TAP1	1.00E-05	-16.61	0.001535	down	Phagosome
A0A8V1ACM1	NCF1C	1.584	0.6636	0.001555	up	Phagosome
P10288	CDH2	0.762	-0.3921	0.002029	down	Cell adhesion molecules
A0A8V1A4B8	PTPRVP	45.48	5.507160349	0.002112	up	Cell adhesion molecules
A0A8V0Z0K5	ITGA11	0.7568	-0.402	0.002239	down	Phagosome
A0A8V0YDM1	NCF2	1.259	0.3323	0.00337	up	Phagosome
A0A8V0YJE5	TFRC	1.394	0.4792	0.003531	up	Phagosome
Q6T7B9	CXCL12	0.7468	-0.4212	0.005623	down	Intestinal immune network for IgA production
Q4ADW0	CD1	0.4293	-1.22	0.006052	down	Phagosome; Cell adhesion molecules
A0A8V1A9Y1	CRPL2	1.753	0.8098	0.006718	up	Cell adhesion molecules
A0A8V0YB58	SELP	1.26	0.3334	0.01052	up	Cell adhesion molecules
A0A8V1A9Q5	GPR182	0.732	-0.4501	0.01057	down	Intestinal immune network for IgA production

Table S3 The top 20 proteins significantly regulated by ANT compared to MOD

Accession	Gene Name	FC	Log2FC	P-value	Regulate	Pathway
A0A1D5PLI8	BLB1	2.608	1.383	0.003266	up	Phagosome; Cell adhesion molecules; Intestinal immune network for IgA production
A0A1D5PM03	PTGS1	1.34	0.4222	0.003586	up	Phagosome
A0A1D5PZB7	NOS2	1.268	0.3426	0.6023	up	Phagosome
A0A290XWB2	TLR2	1.299	0.3774	0.02202	up	Phagosome
A0A2H4C5K1	BF	0.7655	-0.3855	0.2963	down	Phagosome; Cell adhesion molecules
A0A3Q2TU19	EPX	2.398	1.262	1.486e-06	up	Phagosome
A0A3Q2TZZ7	PTPRF	0.8011	-0.3199	0.00464	down	Cell adhesion molecules
A0A3Q2UG72	C4	0.7639	-0.3885	0.2245	down	Phagosome
A0A3Q3A736	COLEC11	0.727	-0.46	0.1717	down	Phagosome
A0A3Q3A741	PTPRE	1.215	0.281	0.08334	up	Cell adhesion molecules
A0A452J7Z2	SDC4	1e-05	-16.61	0.0003895	down	Cell adhesion molecules
A0A8V0X1T8	BCHE	0.7683	-0.3803	0.01775	down	Cell adhesion molecules
A0A8V0XBG4	FNDC3A	0.5365	-0.8983	0.00878	down	Cell adhesion molecules
A0A8V0XC28	CD99	0.5296	-0.917	0.03104	down	Cell adhesion molecules
A0A8V0XFZ5	SCARB2	0.8248	-0.2779	0.008816	down	Phagosome
A0A8V0XH43	C4A	0.7824	-0.354	0.1685	down	Phagosome
A0A8V0XKK4	MMR1L4	0.8193	-0.2875	0.004737	down	Phagosome
A0A8V0XLB8	MHCY38	1.561	0.6425	0.4286	up	Phagosome;Cell adhesion molecules
A0A8V0XN44	CDH5	1.751	0.8082	0.1498	up	Cell adhesion molecules
A0A8V0XPX9	MRC1	0.7678	-0.3812	0.00532	down	Phagosome
